# The Critical Role of Development of the Transversus Abdominis in the Prevention and Treatment of Low Back Pain

**DOI:** 10.1007/s11420-019-09717-8

**Published:** 2019-08-29

**Authors:** Christine Lynders

**Affiliations:** 1 Hospital for Special Surgery, 535 E 70th St., New York, NY 10021 USA; 2 Christine Lynders Physical Therapy, LLC, Kaneohe, HI USA

**Keywords:** low back pain, physical therapy, transversus abdominis

## Introduction

The rising incidence and prevalence of low back pain constitutes a major public health issue in our society today, with widespread economic, physiologic, and psychological costs. In 2015, it was reported that 80% or more of us will suffer from low back pain at some point in our lifetimes [19, 15] and that at any one time 540 million people worldwide are affected by low back pain [6, 7]. It is now the most common cause of job-related disability and a main reason for lost time at work [2, 6, 7].

The causes of this modern epidemic are many, but the sedentary lifestyle of the modern working human—characterized by prolonged periods of sitting and a lack of exercise—is likely a major contributor [4]. It is known that prolonged sitting is detrimental to the maintenance of proper spinal alignment and stability, but mechanical loading of the spine during sitting and standing is still not well understood. A commonly cited 1970 study by Nachemson and Elfstrom found that with standing there is approximately 100-kg compression on the spinal discs and that with sitting these forces increase to approximately 140 kg and rise further to 185 kg with forward bending while seated [18]. Lifting an object from that forward seated position can increase those loads to 220 kg [16, 18]. We have also come to understand that prolonged sitting causes lumbar flexion, reversing normal lordosis and leading to increased compressive, static (segmental) loading over time [3]. Callaghan and McGill also found that sitting results in significantly higher compressive loads in the low back than standing does [3].

Spinal structure is designed to maintain upright posture, absorb shock, and accommodate bipedal gait through three normal curves: cervical lordosis, thoracic kyphosis, and lumbar lordosis. Spinal alignment also depends on stabilizing structures such as the facet joints, spinal ligaments, and the intervertebral discs, as well as the muscles that provide dynamic stability by absorbing the energy of loading the spine during activities. Similarly, many treatments are available to manage chronic low back pain, but nonpharmacologic methods such as exercise are strongly recommended as the initial approaches [21].

The core muscles, consisting of the trunk muscles from the diaphragm to the pelvic floor, provide critical dynamic stabilization for the lumbar spine. Included in this group are the muscles of the pelvic floor, the rectus abdominis, the internal and external obliques, and, most critically, the transversus abdominis, the deepest abdominal muscle. Posteriorly, the erector spinae muscles span multiple levels of the spine and provide for erect posture. The deepest compartment posteriorly consists of the multifidus muscles, which provide segmental stability to individual vertebrae and increase the stiffness of the spine during function [11, 20, 27]. The multifidus muscles work with the transversus abdominis and the pelvic floor muscles to form what is known as the anatomical girdle. These deep compartment muscles are most important to provide essential stability to the spine [10, 11, 20, 23–25].

There is a great deal of support for the concept that the deep compartment muscles are the most critical to stabilizing the lumbar spine segmentally during activity, preventing deleterious loading that can result in injury and pain [20, 27]. Mani et al. reported that muscle injury or fatigue or spinal joint or disc degeneration can compromise the stabilizing effects of the structures in the spine, resulting in shearing forces that can cause pain [15]. They showed that activation of the deep core structures by the abdominal drawing-in maneuver (ADIM) creates a co-contraction between the transversus abdominis and the multifidus, resulting in decreased pain and improved symmetry during walking in those with low back pain [15]. Chon and colleagues in 2010 found that core stabilization techniques involving the recruitment of the central core stabilizer, the transversus abdominis, are necessary for managing low back pain and that the abdominal drawing-in maneuver was more effective at activating the transversus abdominis than general core stabilization techniques [5]. In a comparison study, Bhadauria et al. found lumbar stabilization to be a more effective exercise than Pilates and dynamic strengthening for those with chronic low back pain because it improved transversus abdominis activation and also addressed the multifidus [1].

In my practice, I have found the cue that best recruits deep core stabilization is to “suck it in” from the navel and below. This maneuver is the same as the abdominal drawing-in maneuver, which Mani et al. defined as “an inward movement of the lower abdominal wall in which the patient is instructed to draw the umbilicus toward the spine while maintaining a normal lumbar lordotic curve along with relaxation of the more superficial musculature” [15]. In my experience, most people cannot relate to or visualize the term “draw in.” They can, however, relate to and visualize “suck it in”—almost everyone has sucked in the belly at one time or another.

Regardless of the terminology used to describe it, the benefit of activating the transversus abdominis correctly is achieving a co-contraction with the deepest segmental spinal stabilizer, the multifidus. Patients who would most benefit from it include those who have chronic back pain, those who have had back or abdominal surgery and are cleared by the surgeon to begin strengthening, post-partum women, children or adolescents with scoliosis, athletes, or sedentary individuals. Those who may not benefit include patients who have had abdominal hernia repair, those with severe diastasis recti, or those with severe uncorrected abdominal hernia. Hodges and Richardson in 1996 [10] and Lamoth et al. in 2008 [13] demonstrated that intentional transversus abdominis contraction, while in the neutral spine position (normal backward curve), is associated with lumbar multifidus contraction to activate the anatomical girdle, which augments low back stability [9, 15]. Additionally, Soundararajan and Thankappan found that retraining the deep multifidus was more effective than traditional back exercises for decreasing pain and functional disability in people with chronic low back pain [25].

These principles apply equally to non-athletes and athletes alike. Training the core muscles to stabilize the spine and trunk is essential in all sports activities. Of critical importance is the transversus abdominis muscle, which contracts just after your brain has the initial thought for motion [10–12]. During athletic activity, which involves constant motion and change, the transversus abdominis plays a role in stabilizing not only the trunk but also the extremities. For example, when an athlete raises an arm to strike a ball as in the volleyball serve or spike, the transversus abdominis and multifidus muscles must contract to stabilize the athlete while kinetic energy is transmitted to the ball. Similarly, a soccer player preparing to kick or a tennis player preparing to serve needs a well-functioning, anatomical girdle to avoid undue strain and injury.

## Training the Core

Mastery of the exercises that strengthen the core muscles and lead to lumbar stabilization begins with learning to activate the transversus abdominis. Imagine trying to move your navel closer to your spine or moving your two pelvic bones closer toward each other to engage the transversus abdominis. Another technique is performed by inhaling deeply and then slowly blowing air out through a small hole between your lips while pulling the navel closer to the spine. This action initiates transversus abdominis firing (along with the internal oblique, the pelvic floor muscles, the diaphragm, and the multifidus).

To try this ADIM or “suck it in” technique, lie on your back on a firm surface with your knees bent and the soles of your feet flat on the surface. Pull your navel in so it moves closer to the surface, closer to your spine. Do not bear down or bulge out your lower abdominals. When your transversus abdominis contracts, the belly moves slightly inward, not outward. Do not flatten the back, squeeze the buttocks, or press the small of the back into the surface, which works the superficial abdominals. The preferred technique requires isolating the deep abdominals and not the superficial global muscle groups. Avoid tilting the pelvis, which activates the rectus abdominis, leading to reversal of the normal lordotic curve. We want to maintain the spine’s neutral position or lordosis to engage these muscles properly [13, 17, 22, 26]. To perform this in the standing position, place the left hand just below your sternum and the right hand just beneath the belly button. Correct activation of the transversus abdominis will result in the lower hand moving inward while the upper hand remains stationary.

To many in this field, previous methods to stabilize the spine that focused on the posterior pelvic tilt have failed. Typically, this approach instructed the patient to press the back flat to the floor to engage the abdominal or core muscles. This posterior pelvic tilting, however, takes the spine away from its optimal neutral position and places it into flexion, rendering the deep spinal stabilizers ineffective and increasing activation of the more superficial abdominals [14, 17, 22, 26]. It is my personal clinical experience that the posterior pelvic tilt is not a successful strategy for most patients with low back pain. Furthermore, in my experience, instruction in this neutral spine transversus abdominis–multifidus co-contraction technique eliminates pain, allowing resumption of most activities, including sports.

The next step is incorporating the maneuver into activities of daily living. The key is to keep the lumbar spine in a neutral position, or slight lordosis, while engaging the transversus abdominis to develop an anatomical girdle. Likewise, activation of “suck it in” before bending (at the hips, not the waist) protects the spine with an anatomical girdle of support. Performance of this maneuver before leaning over the sink when brushing the teeth or washing the face also protects the spine. The process of stabilizing the lumbar spine must begin with this re-education and activation of the transversus abdominis and multifidus muscles. First, patients must locate the transversus abdominis muscle contraction by placing the fingertips at the front of the pelvis and sliding them just inward off the bone. Correct activation of the deepest abdominals can be felt by a firmness under the fingertips once the area at and below the navel is pulled inward actively. Next, patients must pay attention to keeping the low back in neutral and not flattening it to the floor. Finally, patients need to strengthen the transversus abdominis contraction by using it during activities of daily living. The goal is to achieve automatic function of the transversus abdominis as it was designed [10–12] to allow healing and prevention of future injury.

## The Therapeutic Program

For appropriate patients, the physical therapy program should focus on the following:

1. Relieve pain and muscle spasm. Use massage, ice, heat, and stretching. Lumbar decompression can be instrumental initially in relieving pain and spasm. Decompression is performed by instructing the patient to lie facing up with the legs flexed over a support such as a box or ottoman. The hip and knees should be in 90–90 flexion, in which the upper leg is perpendicular to the torso and bent 90°. This position should be maintained as long as possible for up to 60 min and can be performed throughout the day to relieve spinal compression and pain, especially after aggravating activities such as prolonged driving or working in a seated position.
2. Start educating the transversus abdominis and multifidus muscles with the “suck it in” maneuver. Focus on correct posture.
3. Progress to core stabilization exercises to protect the spine and incorporate into daily activities.

The foundation of the therapeutic program is the development of correct posture during standing and sitting. To correct standing posture, have the patient begin by standing with the head, upper back, and buttocks (but not the low back) resting against a wall, feet forward 6 to 12 in. The patient should actively squeeze the shoulder blades and press the head back toward the wall, with the chin tucked enough to keep it and the eyes level. Doorway chest stretching can be used to help to stretch the tight chest muscles and allow the patient to be more erect at the wall. This is done by carefully standing in a doorway with arms outstretched at shoulder height. With one foot in front of the other in a lunge position, the patient bends the front knee until a stretch is felt at the front of the chest, just inward of the shoulder joint. Breathing in and out deeply can enhance this stretch. To correct sitting posture, the chest should be aimed upward with the shoulder blades slightly clenched down and back. The hips should be positioned slightly higher than the knees. This can be achieved by moving the feet underneath the chair. A pillow behind the low back can help maintain the lumbar lordotic curve (or neutral spine) to provide the least amount of compression to the discs in sitting.

**Fig. 1:**
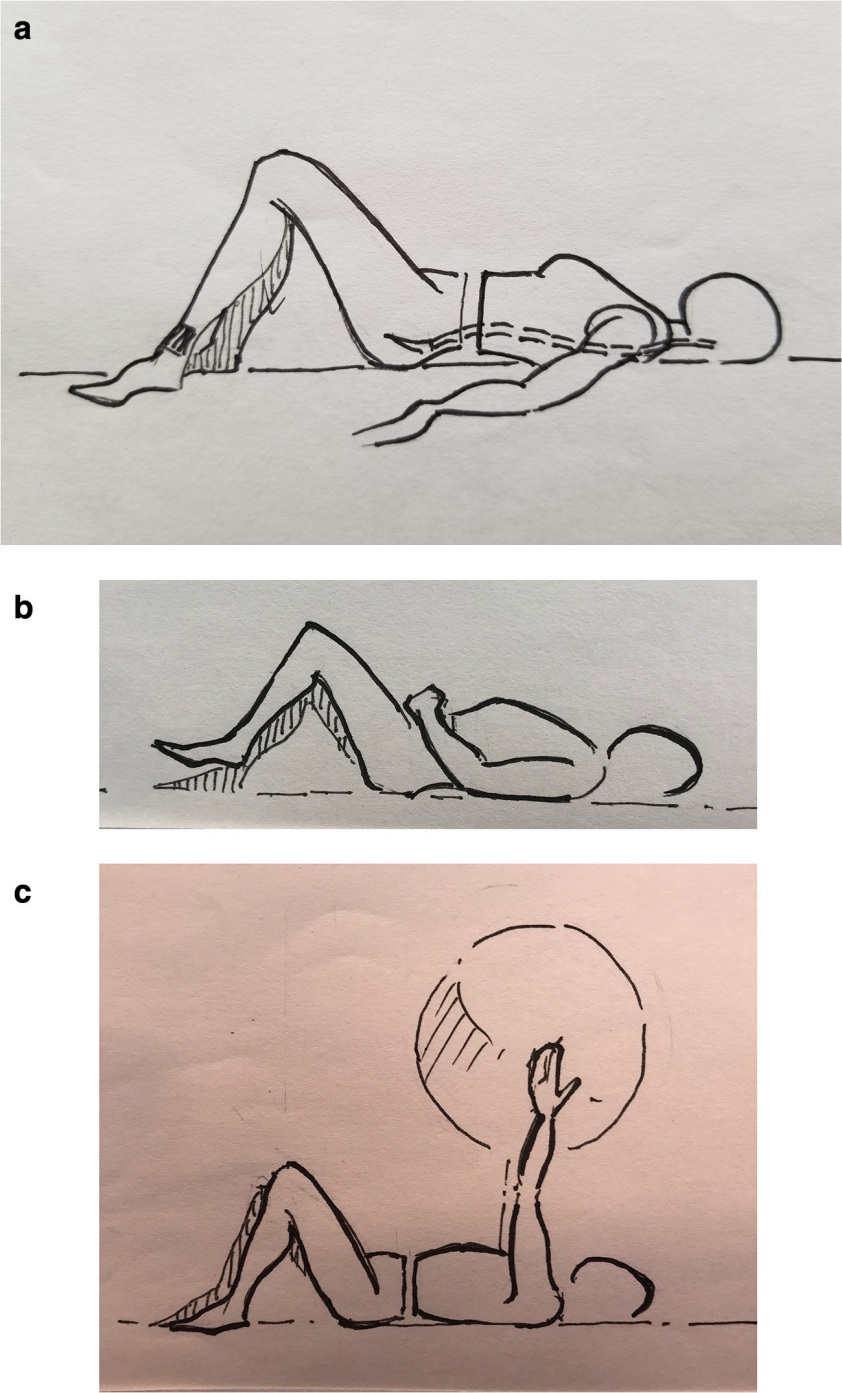
Beginner-level exercises introduced early in the retraining program: **a** transversus abdominis activation with neutral spine, **b** transversus abdominis march, and **c** transversus abdominis activation with ball circles. Illustrations by Joseph F. Lynders and Christine Lynders.

**Fig. 2:**
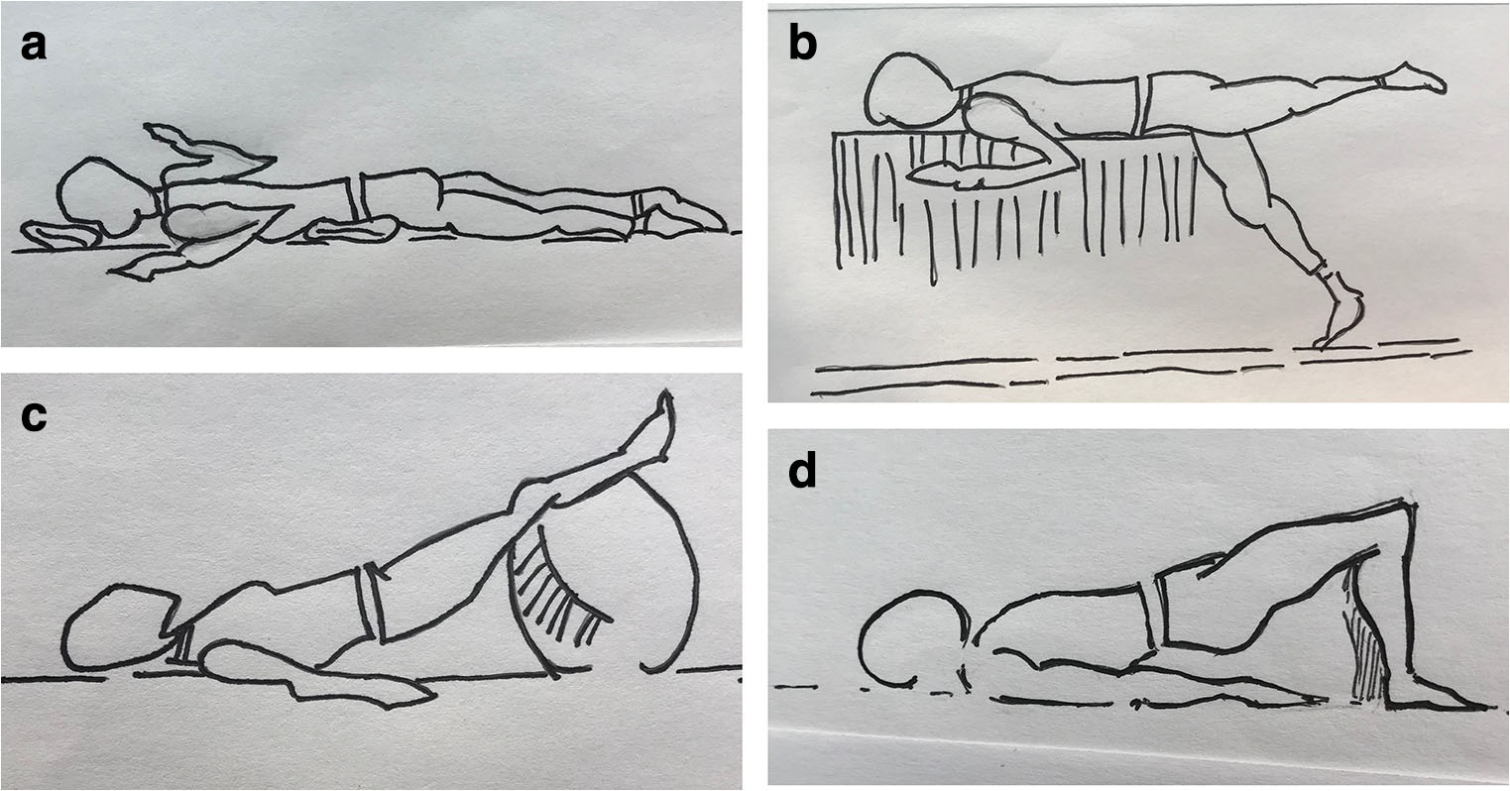
Exercises introduced after the “suck it in” maneuver has been properly achieved with leg movement: **a** prone transversus abdominis “W” scapular retraction, **b** half-prone over table transversus abdominis alternate leg lift, **c** transversus abdominis long leg ball bridge, and **d** transversus abdominis bridge.

**Fig. 3:**
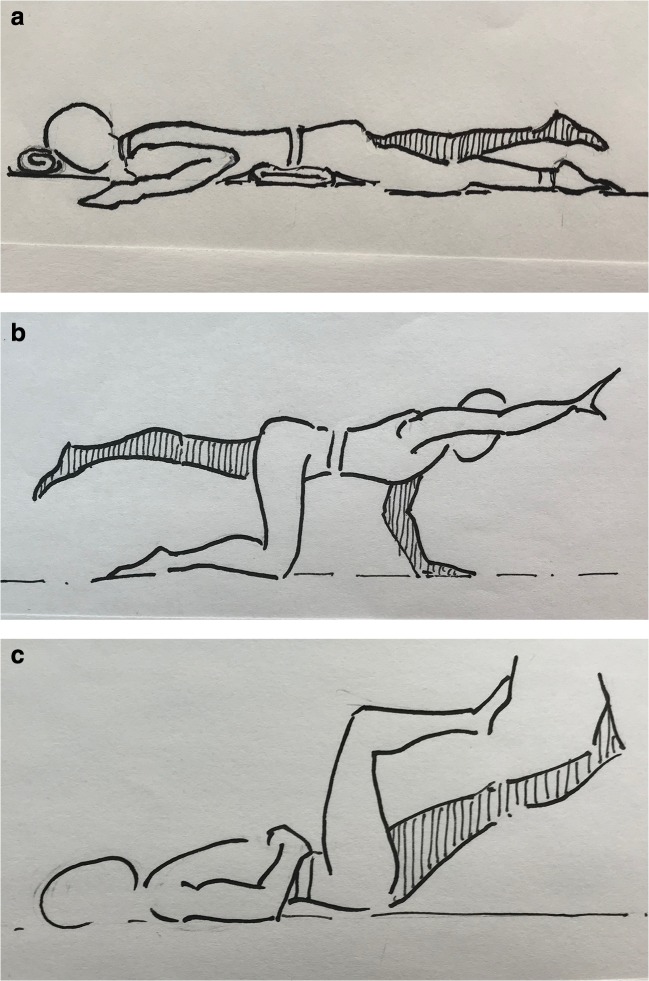
More advanced exercises: **a** prone over pillow alternate leg raises to strengthen the multifidus, **b** quadruped alternate arm and leg with transversus abdominis, and **c** transversus abdominis mountain climber.

You can then address progressive activation and strengthening of the transversus abdominis. The focus here is on developing a stronger contraction in the transversus abdominis muscle to maintain spinal support in the neutral position and help it to better support the spine under greater load without compromising the position of the spine. This can be done by sucking in from the navel and below and holding the contraction while breathing for an extended duration, such as 30 s to 2 min. The patient can then advance to the transversus abdominis march exercise. This exercise increases the demand on the transversus abdominis–multifidus co-contraction to maintain a neutral spine and level pelvis, essentially increasing the resistance; leg motion simulates walking to train the system to operate during upright function. The transversus abdominis march is performed supine, with the knees bent and feet flat on the horizontal surface. With the transversus abdominis contracted, the feet are alternately raised off the floor about 1 in. The pelvis should remain motionless and the low back neutral, rather than pressed down against the surface (Fig. 1). The goal here is to train the transversus abdominis and multifidus to keep the pelvis and spine still and supported while the legs are moved, as would occur during walking. With progress and mastery, additional transversus abdominis strengthening exercises (Figs. 2 and 3) are added to further strengthen and retrain this deep core mechanism.

## Neuromuscular Re-education and Strengthening

The proper functioning of the transversus abdominis is critically important in relieving low back pain, both in the short and long term. This is achieved through specific re-education: learning to fire the transversus abdominis properly so that it can engage the deepest spinal stabilizers, the multifidus and the pelvic floor, in order to protect the low back during movement and restore the original, automatic nature of this function. Hides et al. report that without specific retraining, the multifidus does not regain normal function after one acute episode of low back pain, which can contribute to recurrent episodes [8].

Proper activation of the transversus abdominis can be learned using exercises—both statically, to ensure that a neutral spine position is achieved during re-education, and dynamically, to allow incorporation of the contraction into activities of daily life. This neuromuscular re-education and strengthening process leads to regaining the automatic function of the anatomical girdle and prevents pain and injury to the low back during stationary sitting, during movement, or when the patient is experiencing low back pain. The goal is the proper engagement of the local muscles that specifically provide stability instead of the larger, more superficial muscles. This is accomplished by learning to recruit the proper muscle firing and patterning, then strengthening these muscles, and finally coordinating them to function as a team during whatever activity is performed.

In this program, certain commonly taught exercises are specifically avoided, including those that reverse lumbar lordosis. In particular, the posterior pelvic tilt exercises are not performed because reversal of the lumbar lordosis inhibits the stabilizing co-contractions of the pelvic girdle and may aggravate instability symptoms.

This approach to care will benefit from future research done to further our understanding of the stabilizing effects of contraction of the deep core muscles, as well as the deleterious inhibitory effects of harmful postures, exercises (such as the pelvic tilt), and certain activities.

In summary, I have seen many patients with core weakness who do not have low back pain. But in patients I see who do have low back pain, muscle training reduces pain. Patients need to learn to engage the deep core muscles and activate the anatomical girdle to support the low back and eliminate pain. The patient should keep the spine in a neutral position during daily activities. This can be accomplished through sitting properly and supporting the low back curve with a pillow. Patients with low back pain should also avoid activities that reverse the normal lumbar lordosis such as sitting in low slouchy sofas or prolonged gardening in compromising postures. Exercises that reverse the lumbar curve such as curl-up sit ups should also be avoided. The “suck it in” maneuver activates the body’s anatomical support system and begins the process of retraining the anatomical girdle. When mastered, the maneuver trains the anatomical girdle to fire automatically, providing dynamic spinal stabilization during routine and athletic activities.

## Electronic supplementary material

ESM 1 (PDF 1224 kb)
